# Risk Factors of Incident Kidney Stones in Indian Adults: A Hospital-Based Cross-Sectional Study

**DOI:** 10.7759/cureus.35558

**Published:** 2023-02-27

**Authors:** Smriti Singh, Sanjay Gupta, Tushar Mishra, Basu Dev Banerjee, Tusha Sharma

**Affiliations:** 1 General Surgery, Saint Peter’s University Hospital, Surrey, GBR; 2 General Surgery, University College of Medical Sciences, New Delhi, IND; 3 Surgery, All India Institute Of Medical Science, Bhubaneswar, Bhubaneswar, IND; 4 Biochemistry, Guru Teg Bahadur Hospital, University College of Medical Sciences, New Delhi, IND; 5 Biochemistry, University College of Medical Sciences, New Delhi, IND

**Keywords:** fok1 allele, heavy metal, vdr gene, risk factors of renal stones, renal stones, kidney stones

## Abstract

Background

The diverse manifestations of urolithiasis provide very interesting epidemiological data. This has prompted various studies to look into the etiopathogenesis of renal stones, which is believed to be multifactorial, both exogenous and endogenous. VDR Fok1 is a risk factor for renal stone formation and could cause the formation of renal stones through the mechanism of crystal induction and crystallization in the urine. While a few recent studies have shown the role of heavy metals like cadmium and lead in the formation of renal stones, the current knowledge is still insufficient.

Methods

This case-control prospective study was conducted in Guru Teg Bahadur (GTB) Hospital, a tertiary care facility in Delhi with 30 cases and 30 controls. Patients visiting the department of surgery between November 2011 and April 2013 were enrolled in the study. Cases were defined as patients with renal stones diagnosed on the basis of history and radiological investigations. Controls were selected from the patients admitted to the department of surgery for reasons other than renal stones. The study protocol was approved by the Institutional Ethical Committee of the University College of Medical Sciences, GTB Hospital, Delhi. Written informed consent was obtained from all patients. A structured questionnaire was used to collect data. Metal levels were analyzed by an atomic absorption spectrophotometer (Shimadzu Flame AA-680, Shimadzu Corp., Kyoto, Japan) at Delhi University. The vitamin D receptor gene was measured using genomic DNA. Horizontal agarose gel electrophoresis was used for the quantification of the genomic DNA.

Results

There were 30 cases and 30 controls in the study. Stress was more prevalent among cases (63%) compared to controls (36%). Nearly 83% of cases had the ff allele of the Vitamin D receptor gene compared to 46% of controls. The median arsenic and lead levels were higher among cases compared to controls. In the unadjusted model of logistic regression, we found stressed patients had three times higher odds of developing renal stones compared to non-stressed patients (OR (95% CI): 2.98 (1.04-8.52); p=0.04). Similarly, patients with higher blood concentrations of arsenic and lead had higher odds of developing renal stones compared to those with lower concentrations.

Conclusions

There was a definitive role of heavy metals, including lead, cadmium, and arsenic, seen with renal stones. A significant association was seen between the ff allele of VDR polymorphism (Fok1 enzymes) and patients with renal stones. Other parameters, including male and stress factors, seem to have an important role in renal stone formation.

## Introduction

Around 1% of emergency admissions are caused by renal colic and complications of renal stones [1]. Kidney stones are common across the world, with a prevalence of about 12% worldwide [2]. Their prevalence in India also reflects worldwide prevalence and stands at approximately 12% [3] and is relatively more common in the northern part of India, where it is 15% [1,3]. 

The origin of kidney stones is considered multifactorial, being affected by age, gender, family history, diet, comorbidities, environment, genetic inheritance, and other factors [1]. There are high chances of recurrence of kidney stones irrespective of treatment. It is seen that approximately 98% of patients will develop another stone within 25 years of the first episode [1]. The calcium oxalate variety of renal stones is the most common, constituting 60% of all these stones [4]. 

It has been seen that genetic polymorphism of vitamin D receptor (VDR), Klotho, and chloride voltage-gated channels (CLCN) genes have a role in the formation of kidney stones [4]. VDR is a polymorphic gene having a role in mineral metabolism. It increases the absorption of calcium and excretion of citrate. Studies reveal that multiple allelic variations in VDR, like ApaI, BsmI, TaqI, and Fok1, are associated with nephrolithiasis. VDR Fok1 is a thymine/cytosine polymorphism located at the start (ATG) codon on the 5’ end of VDR, which also showed a role in the formation of renal stones [5].

Another risk factor of renal stone genesis is heavy metals. Heavy metals are a mixed group of elements with metallic properties. Exposure to these heavy metals is mostly via the respiratory or gastrointestinal tracts in the form of cigarette smoke and contaminated food and water. They may cause the formation of renal stones through the mechanism of crystal induction and crystallization in the urine. The toxic manifestations of these metals are primarily due to an imbalance between pro-oxidant and antioxidant homeostasis, which is termed oxidative stress [6]. Cadmium has been a well-known environmental hazard since ancient times [7]. It is used in electroplating, plastics, batteries, mobile phones, and computer circuit boards. In the case of chronic cadmium poisoning, approximately 50% of the accumulated dose is stored in the kidney [7]. B2Micro globulin (b2M) and retinol-binding protein (RBP) in urine are two indicators of early renal toxicity. RBP is said to be the more definitive marker as compared to beta-2 microglobulin, which is also seen in cancer, amyloidosis, and autoimmune diseases like rheumatoid arthritis [7]. As the tubular injury progresses, more generalized tubular dysfunction occurs with wasting and impaired vitamin D metabolism, as well as the reduced conversion of 25-OH vitamin D to 1,25 OH vitamin D and urinary losses of glucose and amino acids, bicarbonate, and phosphate. Cadmium may directly affect bone mineralization, leading to loss of calcium from bone and increased renal excretion. Continued cadmium exposure causes glomerular damage, leading to albuminuria and a progressive decline in glomerular filtration rate (GFR), eventually causing end-stage renal failure [7]. A few recent studies have shown the role of heavy metals like cadmium and lead in the formation of renal stones, although complete knowledge is still deficient [4,7].

In India, few studies are available regarding the risk factors of kidney stones. The present study aims to evaluate various exogenous and endogenous risk factors in patients suffering from renal stones disease.

## Materials and methods

Study population

This case-control prospective study was done in Guru Teg Bahadur (GTB) Hospital, a tertiary care facility in Delhi, India with 30 cases and 30 controls. GTB hospital is a 1700-bed government hospital situated in East Delhi and receives 600 patients in the outpatient department and 250 admissions per day [5]. 

Patients visiting the department of surgery between November 2011 and April 2013 were enrolled in the study. Cases were defined as patients with renal stones diagnosed on the basis of history and radiological investigations (abdominal X-ray or ultrasound abdomen or non-contrast computed tomography of kidney, ureter, and bladder). Controls were selected from the patients admitted to the Department of Surgery for reasons other than renal stones. The study protocol was approved by the Institutional Ethical Committee of the University College of Medical Sciences, GTB Hospital, Delhi. Written informed consent was obtained from all patients. 

Data collection and measurements

A structured questionnaire was used to collect data that had questions on socio-demographic characteristics, such as age, gender, marital status, education, occupation, pregnancy status, present history of illness (symptoms suggestive of renal stones), history of past illnesses (stones, congenital anomaly, systemic diseases, drug intake), family history of stones, and personal history of smoking, alcohol, stress, sleeping, dietary habits (vegetarian or non-vegetarian), and drinking water source, etc. Stress was assessed subjectively by asking a patient if he/she felt it or not, and data was collected as yes/no responses. 

The questionnaire also captured data on anthropometric measurements (height, weight), clinical parameters (pulse, blood pressure), and biochemical parameters (hemoglobin, serum urea, serum creatinine, blood sugar, serum calcium, lead, cadmium, arsenic, and chromium, urine routine microscopy, and genetic markers (Vitamin D receptor gene with FF, Ff, or ff allele). Patients’ radiological investigations such as abdominal X-ray, abdominal/KUB ultrasound, or non-contrast computed tomography of the kidney, ureter, and bladder were also done in the hospital.

Blood sugar levels at fasting, post-prandial, and random state were obtained. Body mass index was calculated from height and weight using the formula.

Height was measured using the stadiometer to the nearest 0.5 cm after removing shoes/slippers and placing heels together, and weight was measured using a standard digital weighing scale to the nearest 0.1 kg while barefoot and in light clothes. Blood pressure was measured using a digital sphygmomanometer available in the hospital. The blood sample was withdrawn and sent to the laboratory outside the hospital (National Accredited Laboratory affiliated with Delhi University) for the measurements of all the clinical parameters.

Heavy metal was estimated by withdrawing 1.5 ml peripheral blood sample in an EDTA vial, 0.5 ml of blood sample was taken in triplicate in a 100 ml digestion flask fitted with a 30 cm long air condenser, and 5.0 ml distilled HNO3 was added to each sample. The contents were heated at 80°C for 30 minutes. After cooling, 1.5 ml of concentrated perchloric acid (70%) was added, and the sample was heated again at 25°C with occasional shaking till white fumes evolved. The clear solution was cooled and transferred into a 10 ml measuring flask. The volume was made up of 10 ml of deionized water [8]. Thus, the obtained sample was filtered by using a syringe filter of 0.45-micron pore size (RFCL Ltd, New Delhi, India), and the metal levels were analyzed by an atomic absorption spectrophotometer (Shimadzu Flame AA-6800, Shimadzu Corp., Kyoto, Japan) at Delhi University.

The vitamin D receptor gene was measured using genomic DNA. Genomic DNA for genotyping was isolated from whole blood by using a commercially available Himedia Hipura blood genomic DNA isolation kit (HiMedia Laboratories Pvt. Ltd., Mumbai, India) as per the manufacturer’s protocol. The DNA was stored at -22ºC till polymorphic analysis was done. The extracted DNA was quantified by taking the optical density (OD) of the sample at 260 nm wavelength by the Shimadzu UV-2450 spectrophotometer. The purity of DNA was ascertained by taking the ratio of OD at 260 and 280nm. The overall purity of the samples (i.e., OD260/ OD280) ranged between 1.3 and 1.7. Horizontal agarose gel electrophoresis was used for the quantification of the genomic DNA. For analysis of VDR polymorphism, PCR-restriction fragment length polymorphism methods were used [9]. A total of 50 ml reaction mixture consisted of 50 ng of genomic DNA, 10 mM of each primer, 0.2 mM of the dNTPs mixture (Bangalore Genei, Bengaluru, India), 1.5 mM of MgCl2, and 1.5 unit of Taq polymerase with 1× PCR reaction buffer (Bangalore Genei).

Primer Sequence for Genotyping of VDR gene

F5’AGCTGGCCCTGGCACTGACTCTGCTCT-3’ and R5’-ATGGAAACACCTTGCTTCTTCTCCCTC-3’.

The restriction enzyme FokI (Fastdigest, Fermentas, Waltham, MA) was used to distinguish the Fok1 polymorphism. The wild-type allele (FF) produced a double band representing the entire 196, 69 base pair (bp) fragment, and the variant allele (ff) resulted in one fragment of 265 bp, whereas the heterozygous allele (Ff) produced all three bands [9]. 

Statistical analysis

Descriptive data were presented as frequencies and percentages for the categorical variables and means/medians with standard deviation or interquartile range for numerical variables. The age variable was categorized into three categories (less than 27 years, 27-45 years, and above 45 years). Chi-square was used to compare the difference in the distribution of independent variables (categorical) between cases and controls. We used the Mann-Whitney test to find a statistical difference between cases and controls in the mean levels of serum arsenic, chromium, lead, cadmium, urinary calcium, uric acid, citrate, blood urea, serum calcium, phosphate, creatinine, serum uric acid, and mean body mass index, and diastolic blood pressure.

All the independent variables that showed a statistically significant difference between cases and controls were put in the regression model. Unadjusted and adjusted models of logistic regression were used to explore associations between independent variables and renal stone history. An odds ratio (OR) with a 95% confidence interval (CI) was used to express the strength of the association. Data were analyzed using Statistical Package for Social Sciences (SPSS) for Windows version 27.0 (IBM Corp., Armonk, NY) with a two‑sided p-value of <0.05, which was considered statistically significant. 

## Results

There were 30 cases and 30 controls in the study. Table 1 shows 60 cases and controls; 42 were males, and 18 were females. There was no statistical difference in the prevalence of history of diseases, smoking, alcohol intake, and drug intake between cases and controls. Stress was more prevalent among cases (63%) compared to controls (36%). In Table 2, nearly 83% of cases had the ff allele of the Vitamin D receptor gene compared to 46% of controls. Table 3 showed no difference in BMI, blood pressure, serum uric acid, serum chromium, serum cadmium, creatinine, serum calcium, and serum phosphate between cases and controls. The median arsenic and lead levels were higher among cases compared to controls. In Table 4, there was no statistically significant difference in the median levels of urinary calcium, urinary citric acid, and urinary uric acid, between cases and controls. Table 5, In the unadjusted model of logistic regression, we found stressed patients had three times higher odds of developing renal stones compared to non-stressed patients (OR (95% CI): 2.98 (1.04-8.52); p=0.04). Similarly, patients with higher blood concentrations of arsenic and lead had higher odds of developing renal stones compared to those with lower concentrations. In the adjusted model, however, most of the associations were insignificant except for stress and the presence of the FF or Ff allele of the Vitamin D receptor gene. Patients with FF or Ff allele had lower odds of developing renal stones than their counterparts (OR (95% CI): 0.15 (0.03-0.80); p=0.02). 

**Table 1:** Distribution of socio-demographic and clinical parameters among cases and controls *Chi-square test was performed

Variables		Cases n=30	Cases in %	Controls n=30	Controls in %	P value*
Age (years)	Less than 27 years	14	46.7	6	20.0	0.09
	27-45 years	9	30.0	13	43.3	
	>45 years	7	23.3	11	36.7	
Gender	Male	22	73.3	20	66.7	0.57
	Female	8	26.7	10	33.3	
Residence (city)	Delhi (Urban)	21	70.0	19	63.3	0.50
	Others	9	30.0	11	36.7	
Marital status	Married	20	66.7	27	90.0	0.03
	Unmarried	10	33.3	3	10.0	
History of drugs intake	Present	6	20.0	2	6.7	0.13
	Absent	24	80.0	28	93.3	
History of diseases	Present	3	10.0	6	20.0	0.28
	Absent	27	90.0	24	80.0	
History of smoking	Present	3	10.0	7	23.3	0.16
	Absent	27	90.0	23	76.7	
History of alcohol intake	Present	7	23.3	5	16.7	0.52
	Absent	23	76.7	25	83.3	
Stress	Present	19	63.3	11	36.7	0.04
	Absent	11	36.7	19	63.3	
Posture of sleep	Supine	13	43.3	21	70.0	0.02
	Right	7	23.3	7	23.3	
	Left	10	33.3	2	6.7	
Diet	Vegetarian	12	40.0	18	60.0	0.12
	Non-vegetarian	18	60.0	12	40.0	

**Table 2:** Distribution of gene variables across cases and controls *chi-square test was performed

Variables		Cases n=30	Cases (%)	Controls n=30	Controls (%)	P-value
Gene	FF and Ff	5	16.7 %	16	53.3%	0.009*
	ff	25	83.3 %	14	46.7%	

**Table 3:** Distribution of body mass index, diastolic blood pressure, concentrations of minerals, and serum concentrations of biochemical parameters across cases and controls †Mann-Whitney test for independent samples

Variables	Cases	Cases median (interquartile range)	Controls	Controls median (interquartile range)	P value
Body mass index	23.6	20.3-26.0	23.9	20.7-27.1	0.79
Diastolic blood pressure	77.0	67.7-86.0	78.0	71.0-85.5	0.59
Serum uric acid	6.0	5.2-6.9	5.9	5.2-6.4	0.79
Serum arsenic	0.89	0.37-1.24	0.31	0.14-0.80	0.03†
Serum lead	2.01	0.4-3.3	0.39	0.1-0.7	0.005†
Serum chromium	0.46	0.2-0.8	0.29	0.2-0.6	0.43
Serum cadmium	0.53	0.1-1.2	0.19	0.1-0.4	0.19
Blood urea	28.0	21.5-32.5	28.0	21.0-32.5	0.79
Serum creatinine	0.8	0.7-1.1	0.8	0.5-0.9	0.79
Serum calcium	8.5	7.5-1.1	8.8	8.1-9.3	0.79
Serum phosphate	3.6	3.1-4.2	3.5	3.2-3.9	0.79

**Table 4:** Distribution of the concentrations of minerals, and urinary concentrations of biochemical parameters across cases and controls †Mann-Whitney test for independent samples

Variables	Cases	Cases median (interquartile range)	Controls	Controls median (interquartile range)	P value
Urinary calcium	200.5	128.2-315.5	211.5	188.7-290.8	0.43
Urinary uric acid	252.0	145.5-335.0	215.0	163.7-287.0	0.19
Urinary citrate	3.0	2.45-3.9	2.7	2.3-3.2	0.19

**Table 5:** Unadjusted and adjusted models of logistic regression to explore associations between independent variables and cases and controls *Control category was the reference category

Variables		Unadjusted* odds ratio (95%CI)	Reference	p-value	Adjusted* odds ratio (95% CI)	Reference	p-value
Marital status	Married/ unmarried	0.22	0.05-0.91	0.03	0.31	0.05-1.75	0.18
Stress	Present/ absent	2.98	1.04-8.52	0.04	5.01	1.09-22.90	0.03
Posture of sleep	Supine/ right/ left	0.32	0.11-0.95	0.04	0.40	0.09-1.74	0.22
Gene	FF or Ff or ff	0.17	0.05-0.58	0.004	0.15	0.03-0.80	0.02
Arsenic		1.82	1.11-2.96	0.01	1.63	0.88-3.03	0.12
Lead		1.92	1.23-2.99	0.004	1.62	0.96-2.71	0.06

## Discussion

Kidney stones are common and potentially preventable causes of morbidity in the general population. The aim of this study was to study the risk factors in patients with renal stones who visited our institution. A total of 30 cases and 30 age- and gender-matched controls were studied. Patients with radiologically diagnosed renal stones were included in the study. 

In the present study, there was a significant difference in the number of males as compared to females with renal stone disease (22 males and eight females, p=0.016). The higher incidence of renal stones in males could be contributed due to the presence of testosterone [1, 6]. A study done in Ballabhgarh Health and Demographic Surveillance System (HDSS) from November 2012 to December 2013 on 433 subjects found that the most common age group affected was 20-40 years; many from this age group are a part of the workforce, which increases the burden on the family and society [10]. Another study published in 2021 also showed that the mean age of renal stone occurrence was 21-40 years [1]. A retrospective study on 435 patients who visited the urology outpatient clinic in Dehradun, India, between 2005 and 2018 showed a high prevalence of renal stones in males, which was almost three times higher than in females. It was also suggested that this prevalence might be due to high levels of testosterone in males [6]. Another study done by Lohiya et al. in northern India, which included 435 renal stones patients, demonstrated a high prevalence of stones in males, which was 1.5 times more than in females [10]. A study done by Semins et al. in approximately 3.4 million insured individuals during a five-year period (2002 to 2006) concluded that the occurrence of renal stones is directly proportional to age, and the disease was twice more common in males as compared to females [11]. Another study done in three cadmium-contaminated villages revealed that there were significant differences between age, gender, alcohol consumption, body mass index, urinary cadmium, and prevalence of diabetes and urinary stones [12].

We were unable to find any association between the risk of urolithiasis with the use of medications and systemic diseases. It has been said that long-term medications, which are possible risk factors for kidney stone formation, include indinavir, thiazide, loop diuretics, thyroid hormones, and antacids. Indinavir stones are seen in 4-12% of treated patients. The possible mechanism of indinavir stone formation is poor solubility and high urinary excretion of this drug. Thiazide causes hypocitraturia whereas loop diuretics cause hypercalcemia [13]. Hyperparathyroidism increases the risk of kidney stone disease by increasing the excretion of calcium, secondary to excessive resorption of bone (known as resorptive hypercalciuria) [14]. Diseases including diabetes mellitus, hypertension, urinary tract infection, gout, inflammatory bowel disease, chronic diarrhea, and cancers like leukemia and lymphomas are associated with the formation of renal stones. In diabetes, the incidence of uric acid stone formation is high due to insulin resistance, a decrease in urinary pH, and obesity which favors uric acid and mixed urate-calcium oxalate stone formation [15]. In hypertension, there is low excretion of citrate and high uric acid excretion along with high BMI, which predisposes to the formation of renal stones [16].

No significance was found between alcohol intake and diet changes with the risk of getting stones. The incidence of renal stone formation increases with a higher intake of alcohol due to an increase in serum uric acid levels [17]. Dietary habits play an important role in the formation of stones. In countries like India, changing lifestyle has increased the tendency for obesity. A fatty diet is thought to create a predisposition to renal stone formation [18].

In the present study, different allele patterns of the VDR (Fok1) gene were found. The incidence of the “ff” allele was significantly higher (p=0.009) in renal stone patients as compared to controls. However, no definitive consensus can be drawn by analyzing previous studies on the higher occurrence of renal stones related to the ff genotype of the VDR gene. Another case-control study on 60 adults aged between 18-90 years showed that VDR and CLDN genes are associated with recurrent urolithiasis. This study also emphasizes the role of methylation of genes in the genesis of renal stones [19]. Another study done in 2015 that included 105 individuals with renal stones showed a significant association of urolithiasis with the FOK1 f allele, which could be directly contributed to the presence of Randall plaques (p=0.047) [20]. In a study published in 2005 by Bid et al. from Sanjay Gandhi Postgraduate Institute of Medical Sciences, Lucknow, the association of vitamin D receptor-gene (Fok1) polymorphism with calcium oxalate nephrolithiasis was studied in 138 patients of calcium oxalates stones and 166 healthy patients. The study found a significant association between renal stones and VDR Fok1 polymorphism (p<0.001) [9]. A meta-analysis done by Lin et al. included 17 studies to explore the association between VDR polymorphic sites ApaI, BsmI, TaqI, and Fok1 and urolithiasis risk. This meta-analysis suggested that in the Asian subgroup, there is an increased risk of urolithiasis with the ff + Ff genotype, and VDR polymorphisms could be potential biomarkers for urolithiasis susceptibility [21].

Metabolic factors including lithogenic conditions such as hypercalciuria, hyperuricosuria, hypercalcemia, hyperoxaluria, hyperphosphaturia, hypocitraturia, the excess load of lithogenic substances like vitamin D, and urinary phytate levels have been implicated as risk factors in kidney stone formation. Hypercalciuria causes supersaturation of urine, which leads to the development of renal stones [22]. Hyperuricosuria is an important metabolic risk factor in the formation of uric acid stones. The etiologic mechanisms for uric acid stone formation are diverse and include congenital, acquired, and idiopathic causes. Major factors for the development of uric acid stones are low urine volume, acidic urine pH, and hyperuricosuria. However, abnormally acidic urine is the principal determinant in uric acid crystallization [22]. Hypocitraturia, with a urinary citrate level of less than 320 mg/day is another important and correctable metabolic cause of renal stones. It is seen in 10% of patients having calcium stones [14]. Our study didn’t find a strong correlation between metabolic disease and the incidence of renal stones. 

Exposure to heavy metals from cigarette smoke and food from contaminated soil and water. The kidney is the main target organ for these heavy metals. Surprisingly, not too many studies have been done on this topic. A recent study published in 2021 done in China on non-occupational exposure found 3.16 times the odds of renal stones with a high level of lead in blood (>100μg/L) in males; however, the odd ratio became 3.43 in the presence of high cadmium in urine and high-level leads in blood. In females, there is a significant correlation between nephrolithiasis and a combination of both high blood lead and urinary cadmium (OR 2.58) [23]. A study was done on 6,748 individuals with exposure to environmental cadmium. The study population was screened for urinary cadmium and calcium levels and the presence of urolithiasis. A strong association was found between urinary cadmium levels and stone prevalence after adjusting other co-variables. The stone prevalence increased 1.093-fold for every 1 µg increase in urinary cadmium when adjusted for urinary creatinine. It was also found that urinary calcium levels increased parallel to increasing urinary cadmium, and this was possibly the mechanism by which cadmium resulted in urinary stone formation [12].

Another study published in 2007 by Bazin et al. suggested the role of heavy metals in stone formation through crystal induction, and the highest proportion of the heavy metals in calcium stones was observed for Zn (mean ± SD= 525 ± 768ppm), followed by Sr (239 ± 300 ppm), Fe (35 ± 43 ppm) and Pb (19 ± 27 ppm) while the other metals accounted for less than 10 ppm on average [6]. A study done on factory workers suggested that an increase in cadmium dose was associated with multiple renal tubular functional abnormalities, including a decrease in resorption of beta-2 microglobulin, retinol-binding protein (RBP), calcium, and phosphate and a rise in mean systolic and diastolic blood pressures. The study also suggested that serum creatinine concentration also increased with an increase in cadmium dosage due to impaired glomerular function. Serum cadmium concentration was significantly higher in the exposed workers than in the unexposed (7.9 v 1.2 µg/l, p< 0.0001). The average blood lead concentration was higher in cadmium workers than in the unexposed (11.9 v 8.3 pg/l, p = 0.0013) [24]. A prospective study by Järup and Elinder on cadmium-exposed battery workers showed similar results [25]. 

In our study, we estimated the serum levels of heavy metals by atomic absorption spectrometry. The serum levels in the patients (cadmium 0.73±0.65; lead 10.29±4.54; arsenic 0.923±0.71ng/ml) were significantly higher than the corresponding levels in the controls (cadmium 0.324±0.27; lead 0.574±0.51; arsenic 0.371±0.29 ng/ml). However, no significant difference was seen in serum levels of chromium in cases and controls.

Even in 2022, we don’t have much information regarding the correlation between psychological stress in the formation of renal stones. A study done by Miyaoka et al. on 200 patients with nephrolithiasis concluded that high stress is related to the stones (p = 0.012) and recurrence of excretion of urinary stones(p=0.022). The author used the PSS-10 score for stress level analysis, and females and unemployment were the confounding factors [26]. Another case-controlled study done at the Center of New Jersey in Newark with 200 individuals matched with age, sex, and race found that three important factors, such as low total annual family income, mortgage issues, and emotional life events that last for more than a week were associated with a higher risk of formation of renal stones (p <0.05) [27].

Strengths and limitations of the study

This study opens a newer perspective to studying the relationship between the FOK1 VDR gene and heavy metals with renal stones. However, this study was conducted in a selected hospital in Delhi, and the sample size was also small; therefore, its generalizability is limited. There was no stress scale used in this study and individual perceptions of stress might be different for different individuals.

## Conclusions

In this case-control study, we studied various risk factors in the formation of renal stones in 30 patients with renal stones (with 30 controls) who visited GTB Hospital, New Delhi. There was a definitive role of heavy metals, including lead, cadmium, and arsenic, seen with renal stones (more with lead). A significant association was seen between the ff allele of VDR polymorphism (Fok1 enzymes) and patients with renal stones. Other parameters, including male gender and stress factors, seem to play an important role in renal stone formation. We conclude that people visiting the outpatient department or admitted to wards should be counseled on these risk factors to prevent the incidence or recurrence of renal stones.
